# Simultaneous Determination of Five Phenolic Acids and Four Flavonoid Glycosides in Rat Plasma Using HPLC-MS/MS and Its Application to a Pharmacokinetic Study after a Single Intravenous Administration of Kudiezi Injection

**DOI:** 10.3390/molecules24010064

**Published:** 2018-12-25

**Authors:** Peiying Shi, Chunlei Yang, Ya Su, Liying Huang, Xinhua Lin, Hong Yao

**Affiliations:** 1 Department of Traditional Chinese Medicine Resource and Bee Products, Bee Science College, Fujian Agriculture and Forestry University, Fuzhou 350002, China; peiyshi@126.com; 2 Department of Pharmaceutical Analysis, School of Pharmacy, Fujian Medical University, Fuzhou 350122, China; yclylf@sina.cn (C.Y.); 15980221531@sina.cn (Y.S.); hlyhly64@163.com (L.H.); xhlin1963@sina.com (X.L.)

**Keywords:** high performance liquid chromatography-tandem mass spectrometry, phenolic acids, flavonoid glycosides, pharmacokinetics, Kudiezi injection

## Abstract

This study has developed a reliable and precise high performance liquid chromatography-tandem mass spectrometry method for the simultaneous determination of five phenolic acids and four flavonoid glycosides in rat plasma after a single intravenous administration of Kudiezi injection (KI). Chromatographic separation was carried out on an Ultimate^®^XB-C_18_ column (4.6 × 100 mm, 3.5 μm) using a gradient elution program with a mobile phase consisting of water containing 0.5% acetic acid and acetonitrile at a flow rate of 0.6 mL/min. Detection was performed on a triple-quadrupole tandem mass spectrometry using multiple reaction monitoring in negative electrospray ionization mode. The calibration curves of all analytes showed good linearity (R^2^ > 0.990). The results of selectivity, intra-day and inter-day precisions, extraction recoveries, matrix effects and stability were satisfactory. Pharmacokinetic parameters showed that luteolin-7-*O*-β-d-gentiobioside, luteolin-7-*O*-β-d-glucuronide, luteolin-7-*O*-β-d-glucoside and apigenin-7-*O*-β-d-glucuronide were eliminated quickly (0.07 h < *t*_1/2_ < 0.66 h), whereas 5-caffeoylquinic acid, caftaric acid, chlorogenic acid, 4-caffeoylquinic acid and caffeic acid were eliminated relatively slowly (2.22 h < *t*_1/2_ < 6.09 h) in rat blood. The pharmacokinetic results would be valuable to identify bioactive constituents, elucidate mechanisms of pharmacological actions or adverse drug reactions and guide the rational clinical use of KI.

## 1. Introduction

Kudiezi injection (KI) is a traditional Chinese medicine (TCM) injection made from *Ixeris sonchifolia* (Bunge) Hence, and its major components are phenolic acids, flavonoids, nucleosides and sesquiterpene lactones [1,2,3]. KI has the pharmacological effects of anti-acute myocardial ischemia [4,5], anti-cerebral ischemia [6,7], anti-hyperlipidemia [8] and so on. KI is mainly used for the treatment of coronary heart disease, angina pectoris, myocardial infarction, cerebrovascular diseases and other diseases in China [9,10,11,12]. Despite favorable therapeutic effects, adverse reactions of KI have also been reported. These include anaphylactic shock, palpitation, vomiting, chill, skin itch and rash, and the analysis results of the influence factors showed that the occurrence of the adverse drug reaction (ADR) could be related to the maximum blood plasma concentration of, the duration of medication in the blood circulation and the dosage of the drug [13]. Thus, understanding the in vivo pharmacokinetic and disposition properties of KI components and formulating the rational dosage regimens are very important.

As we known, in order to determine the pharmacokinetics (PK) of TCMs, it is necessary to measure the fate (including absorption, distribution, metabolism and excretion) of the active components or constituents in TCMs, single TCM or TCM formulas administered to a living organism based on the dynamic principle [14], which could reveal the in vivo disposition of TCMs, and has become one of the important research contents of modernization of TCMs. Additionally, considering that TCMs contain many compounds belonging to different structural categories to produce their therapeutic effects or adverse reactions, the simultaneous determination of multi-components of TCMs in bio-samples and application to PK study are of great significance.

Currently, pharmacokinetic studies of KI have mainly focused on single or a few components. A liquid chromatography-mass spectrometry (LC-MS) method for the determination of luteolin-7-*O*-β-d-glucoside in rat plasma was developed and applied to the pharmacokinetic study of this flavonoid glycoside in rat after intravenous administration of KI [15]. Then, an ultra-fast liquid chromatography-tandem mass spectrometry (UFLC-MS/MS) method for the simultaneous determination of luteolin-7-*O*-gentiobioside, luteolin-7-*O*-β-d-glucoside and luteolin-7-*O*-β-d-glucuronide in beagle dog plasma was established by the same research group, and applied to a pharmacokinetic study of the three flavonoid glycosides in beagle dog after intravenous administration of KI [16]. Moreover, another LC-MS/MS method for the simultaneous quantitation of four flavonoid glycosides, including luteolin-7-*O*-β-d-glucuronide, luteolin-7-*O*-β-d-glucopyranoside, luteolin-7-*O*-β-d-glucopyranosyl-(1→2)-β-d-glucopyranoside, apigenin-7-*O*-β-d-glucuronide and one phenolic acid, chicoric acid in rat plasma, was developed and applied to a pharmacokinetic study of the five analytes in rat after a single intravenous dose of KI [17]. These studies have provided meaningful data for pharmacokinetic profiles of KI. However, despite these components, other main compounds in KI, such as chlorogenic acid, caffeic acid, neochlorogenic acid (5-caffeoylquinic acid (5-CQA)), etc. [18], also possess anti-cardiovascular disease effects [19,20,21,22,23], which should also be determined in biological samples and applied to PK study of KI.

**Figure 1:**
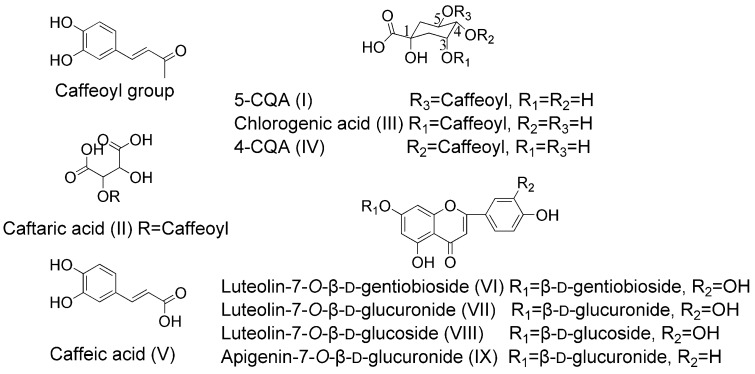
Chemical structures of the nine compounds.

Therefore, in this study, the quantitative analysis method of five phenolic acids, including 5-CQA (I), caftaric acid (II), chlorogenic acid (III), 4-CQA (IV), caffeic acid (V) and four flavonoid glycosides, including luteolin-7-*O*-β-D-gentiobioside (VI), luteolin-7-*O*-β-d-glucuronide (VII), luteolin-7-*O*-β-d-glucoside (VIII) and apigenin-7-*O*-β-d-glucuronide (IX) in rat plasma using HPLC-MS/MS was established and applied to pharmacokinetic study of KI in rats, which could reveal the in vivo process of KI and promote its clinical rational use. The structure of these nine compounds is shown in Figure 1.

## 2. Results and Discussion 

For the LC-MS/MS bioanalysis, a method validation was performed referring to selectivity, linearity, the lower limit of quantification (LLOQ), precision, accuracy, extraction recovery, matrix effect and stability for the nine compounds in plasma.

### 2.1. Method Validation

#### 2.1.1. Selectivity

**Figure 2:**
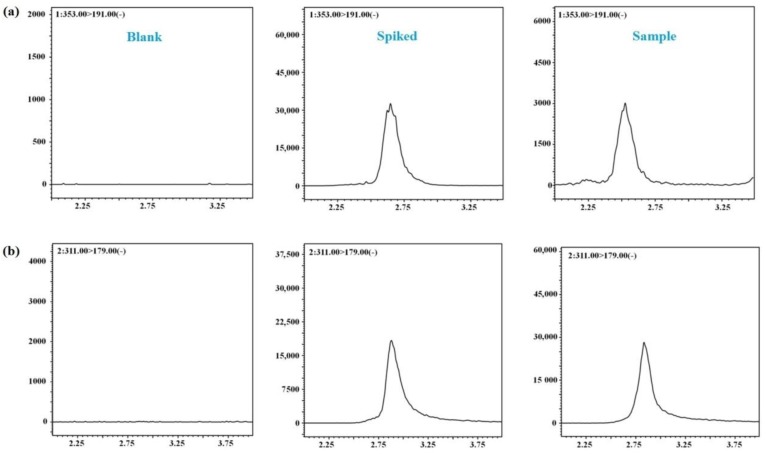

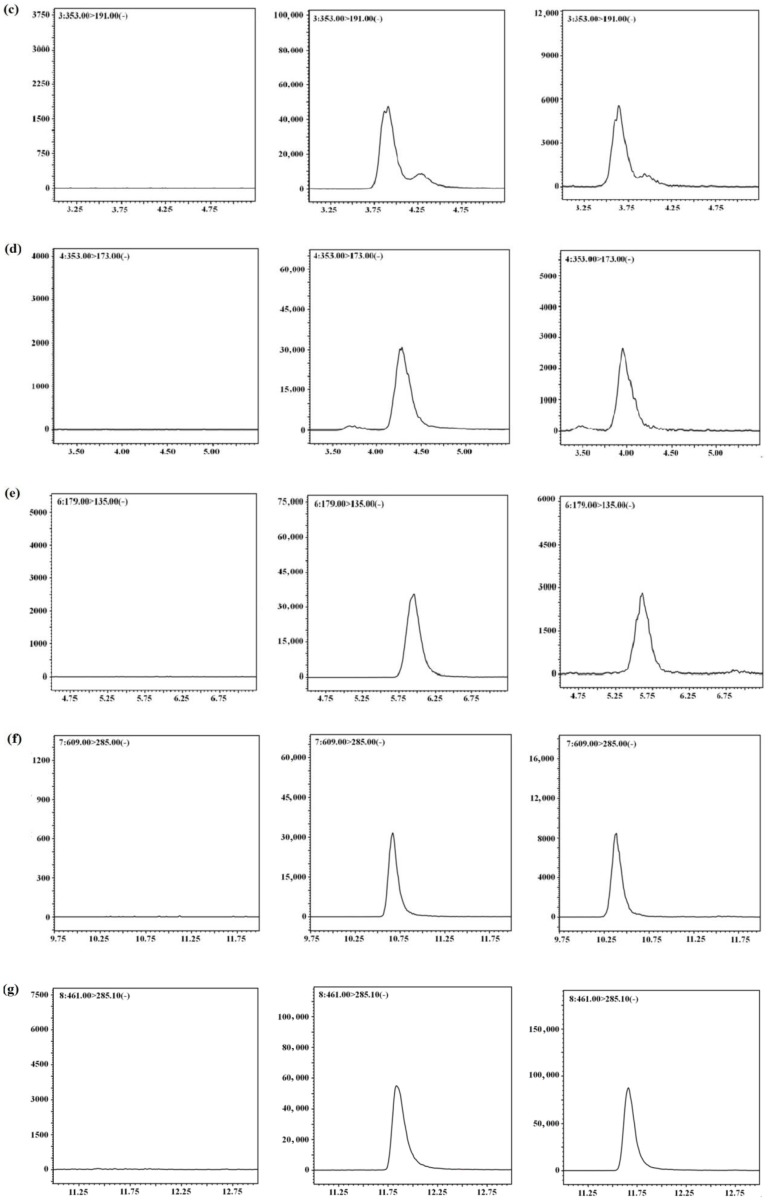

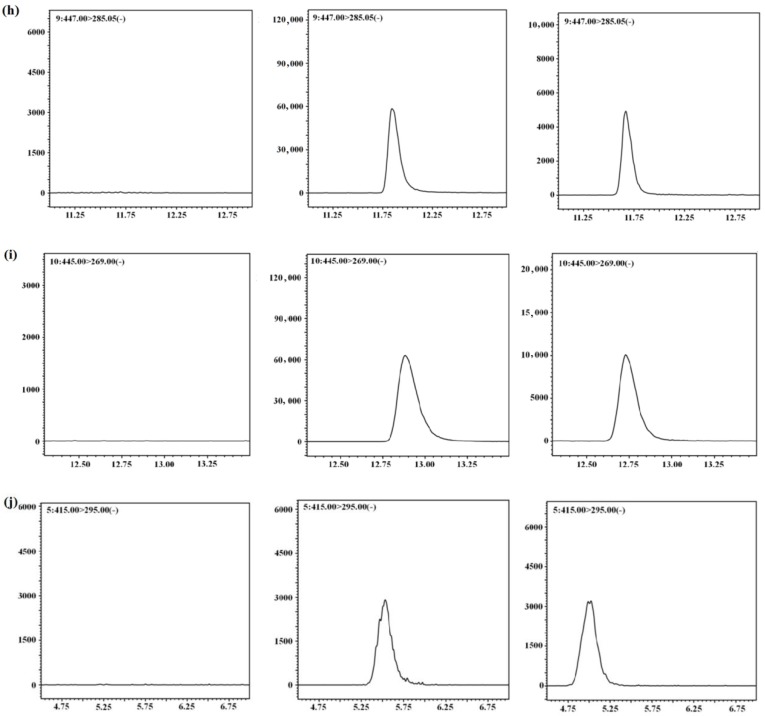
The multiple reaction monitoring (MRM) chromatograms of nine compounds and internal standard (IS) in negative mode. **a**, I; **b**, II; **c**, III; **d**, IV; **e**, V; **f**, VI; **g**, VII; **h**, VIII; **i**, IX and **j**, IS.

The typical multiple reaction monitoring (MRM) chromatograms of the blank plasma sample, the blank plasma spiked with the nine compounds and puerarin (internal standard (IS)) and plasma samples obtained 5 min after intravenous administration are presented in Figure 2, from which no interference or endogeneity could be observed, suggesting good selectivity for the presented LC-MS/MS bioanalysis method.

#### 2.1.2. Calibration Curves and Linearity

The regression equations, linearity ranges and LLOQ for the nine analytes are summarized in Table 1. All calibration curves were of good linearity with a high correlation coefficient (R^2^ > 0.990) over the tested ranges. 

#### 2.1.3. Accuracy and Precision

The results of the intra- and inter-day precision and accuracy of the nine analytes in quality control (QC) samples were shown in Table 2. For all the nine components, relative standard deviation (RSD) values of the intra-day precision were 0.45–5.01% for middle and high concentrations of QC (MQC and HQC) samples and 3.34–18.97% for low concentration of QC (LQC) samples, as well as those of the inter-day precision were 2.37–15.39% for MQC and HQC samples and 5.30–20.49% for LQC samples. Moreover, relative error (RE) values of the intra-day precision were −12.08–10.26% for MQC and HQC samples and from −19.74% to −3.61% for LQC samples. The values of the inter-day precision were −15.69–3.21% for MQC and HQC samples and −20.35–3.13% for LQC samples. Therefore, these results demonstrated that the method was accurate and reliable.

#### 2.1.4. Extraction Recoveries and Matrix Effects

As shown in Table 3, the overall extraction recoveries of the nine compounds were in the range of 68.01–118.98% with RSD values of MQC and HQC samples <14.52% and the RSD values of LQC samples <18.57%, which indicated that the recoveries of the nine analytes were precise and reproducible at different concentration levels in plasma biosamples. Moreover, the RSD values of the matrix effects for MQC and HQC samples are less than 13.14%, and those for LQC samples are less than 18.53%. These results illustrated that the developed method was reliable for bioanalysis.

#### 2.1.5. Stability

The stability of the nine components in rat plasma was investigated under a variety of storage and process conditions. Table 4 summarizes the results of post-preparation (The QC samples were kept in the auto-sampler for 6 h), freeze and thaw and long-term (at −80 °C for 14 days) stability experiments. The results of the stability assay indicated that these analytes were all stable with accuracy (expressed as RE) in a range from −15.00% to 11.47% for MQC and HQC samples and from −18.29% to 1.30% for LQC samples.

#### 2.1.6. Pharmacokinetic Study

**Figure 3:**
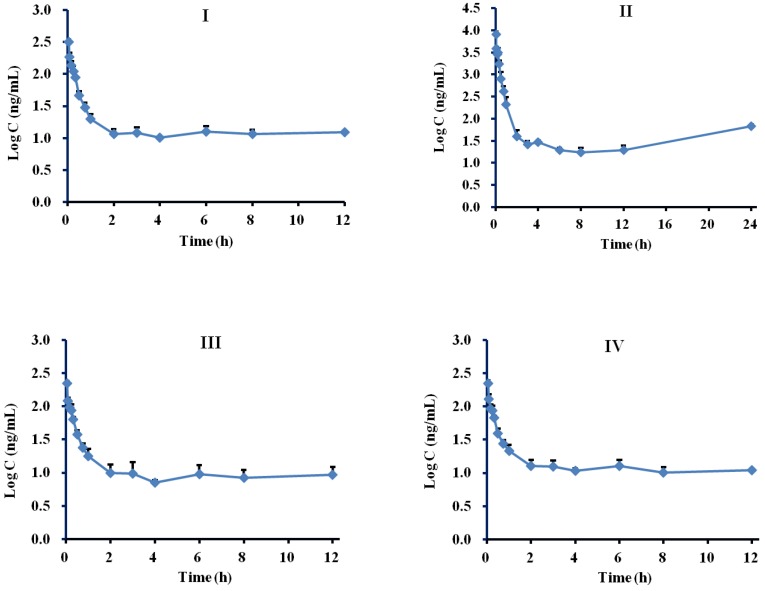

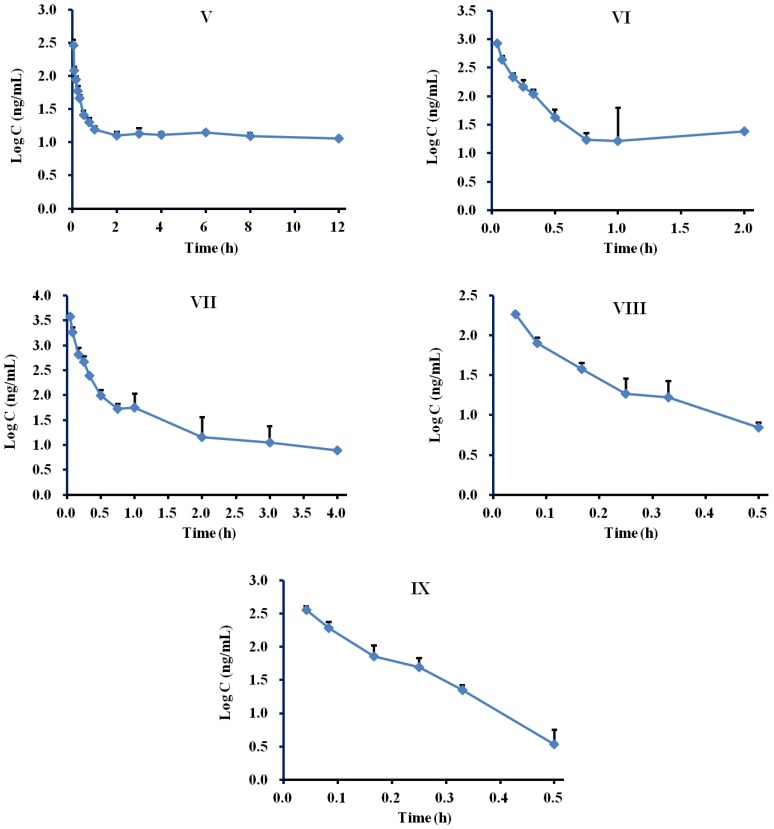
The mean drug plasma concentration-time curves of nine compounds after intravenous administration of 3.6 mL/kg of concentrated KI.

The method validation data were satisfactory, suggesting that the developed HPLC-MS/MS bioanalysis method could be used for the simultaneous determination of nine compounds in rat plasma. The plasma drug concentration-time profiles of the nine compounds after a single intravenous administration of 3.6 mL/kg of concentrated KI are shown in Figure 3.

The respective pharmacokinetic parameters are listed in Table 5. The results showed that the four flavonoid glycosides, including compounds VI, VII, VIII and IX, were eliminated quickly (0.07 h < *t*_1/2_ < 0.66 h), whereas the five phenolic acids, containing compounds I, II, III, IV and V, were eliminated relatively slowly (2.22 h < *t*_1/2_ < 6.09 h) in rat blood. According to the *t*_1/2_ mean values, these ingredients could be ordered as following: V > II > III > IV > I > VII > VI > VIII > IX. Meanwhile, the values of AUC_0–t_ and AUC_0-∞_ of compound II are the largest in the blood circulation of rats, and those of compound VII are the second. The ordering of AUC values for these ingredients is II > VII > I ≈ V ≈ IV ≈ III ≈ VI > IX > VIII. In addition, the Vd, MRT_0-t_, MRT_0-∞_ values of the phenolic acids are greater than those of the flavonoids, while the Cl values of the phenolic acids are lower than those of the flavonoids, suggesting that the phenolic acids stay longer in vivo than the flavonoids. All these results demonstrated that compounds II and VII had a higher systematic exposure to rats. Especially with the longer *t*_1/2_ (3.93 ± 0.47 h) combined with the high in vivo exposure extent, compound II could be speculated to be a main effective substance for KI.

### 2.2. Discussion

Previous studies have reported that the composition of TCMs are complex, and the pharmacological actions of TCMs are thought to result from the synergistic effects of multiple ingredients [24,25]. In this article, the main components, including five phenolic acids and four flavonoid glycosides, were simultaneously determined in rat plasma using a new HPLC-ESI-MS/MS method, and this method was applied to the pharmacokinetic study after a single intravenous administration of KI. These phenolic acids and flavonoid glycosides belong to antioxidants [26,27], which may at least in part prevent atherosclerosis and cardiovascular diseases [28,29]. Especially the phenolic acids, such as compounds I and V having beneficial effects on cardiovascular diseases via suppressing P-selectin expression on platelets [23], compounds III and V possessing anti-myocardial ischemia effects [19,20,21,22], etc., have played important roles in anti-cardiovascular diseases. In addition, PK parameters showed that these active phenolic acids remained unchanged for a longer period in the systemic circulation, suggesting that these phenolic acids exert effects for a long time. Furthermore, compound V stayed longest in vivo, and one of the reasons could be that other phenolic acids, such as compound III, could be metabolized into compound V [30,31].

On the other hand, ADR reports related to TCM injections account for >50% of total ADR reports related to TCM preparations, and most of the frequently reported TCM injections are anti-cardiovascular disease TCM injections, such as Shenmai injection, Xuesaitong injection, Danshen injection, etc. [32]. The ADRs of KI have also been reported, and the data showed that 53.33% of the ADRs, consisting of anaphylactic shock, palpitation, vomiting, chill, skin itch or rash, happened within 30 min [13]. A recent study has identified that luteolin-7-*O*-glucuronide (VII), apigenin-7-*O*-glucronide (IX) and luteoloside (luteolin-7-*O*-glucoside, VIII) could be the potential anaphylactoid components in KI [33]. Although in our study, PK parameters showed that these three flavonoid glycosides stayed shorter in vivo, they could be still related to the ADR of KI. For addressing these problems, more experiments are required.

## 3. Materials and Methods

### 3.1. Materials and Reagents

KIs were produced by Shenyang Shuangding Pharmaceutical Co., Ltd. (Shenyang Shi, China, batch No. 17010701) according to a Chinese patent (CN201110202166.8). The contents of main components in KI were determined by HPLC-UV, including 10.34 µg/mL of I, 214.88 µg/mL of II, 9.12 µg/mL of III, 6.64 µg/mL of IV, 8.86 µg/mL of V, 24.44 µg/mL of VI, 70.61 µg/mL of VII, 4.72 µg/mL of VIII, and 10.29 µg/mL of IX.

Reference compounds I-V, VII-IX and IS were provided by Shanghai Ronghe Medicine Technology Development Co., Ltd. (Shanghai, China). Compound VI was prepared in our laboratory and identified by NMR and MS. The purities of all the compounds were determined to be above 98% by HPLC analysis. Acetonitrile and methanol were of chromatographic grade (Sigma, Kanagawa Prefecture, Japan). Ultra pure water was purified by the Milli-Q system (Millipore, Bedfoed, MA, USA). Glacial acetic acid was purchased from Aladdin Chemical Reagent Co., Ltd. (Shanghai, China).

### 3.2. Animals

Eight male Sprague-Dawley rats (200 ± 20 g) were obtained from Laboratory Animal Center of Fujian Medical University (Fuzhou, China). The animal studies were conducted in accordance with the Guide for the Care and Use of Laboratory Animals published by the USA National Institutes of Health (NIH Publication No. 85-23, revised 1996) and were approved by the Animal Ethic Review Committee of Fujian Medical University (Fuzhou, China). The rats were housed in rat cages (48 × 29 × 18 cm^3^), in a unidirectional airflow room at a temperature of 22 ± 2 °C, a relative humidity of 40–70% and a 12 h light/dark cycle. They were fed with freely available commercial food and filtered tap water, and fasted with free access to water for 12 h before the experiments. Three of these rats were selected randomly as blank group with the above mentioned feeding conditions and without administration of drug, and after 24 h from the beginning of the experiment, their blood was collected up exhaustively from abdominal aorta to provide blank plasma for methodological validation. The rest of the rats (*n* = 5) received a single intravenous administration of 15 times concentrated KI via the tail vein at a dose of 3.6 mL/kg of concentrated KI. All rats were acclimated to the facilities and environment for 7 days before the experiments.

### 3.3. HPLC-MS/MS Instrumentation and Conditions

Shimadzu LC-MS 8040 (Shimadzu, Japan) equipped with an electrospray ionization (ESI) source was used for LC-MS/MS analysis. Separation was carried out by elution on an Ultimate^®^XB-C_18_ column (4.6 × 100 mm, 3.5 μm). The mobile phase consisted of 0.5% acetic acid aqueous solution (A) and acetonitrile (B). The gradient elution was employed as follows: 13–13.5% B at 0–6 min; 13.5–40% B at 6–13 min; 40–95% B at 13–13.1 min and maintained at 95% B from 13.1 min to 15 min. The flow rate was 0.6 mL/min. The column temperature was kept at 30 °C and the volume of sample injected was 5 μL.

The MS conditions for MS/MS were as follows: capillary voltage, 4.5 kV; block heating temperature, 400 °C; desolvation line temperature, 250 °C; dry gas (nitrogen), 12 mL/min; and auxiliary gas (nitrogen), 3 mL/min. MS/MS spectra were obtained for selected precursor ions through collision-induced dissociation with neutral gas (argon) molecules in the collision cell. Quantification was performed using MRM in negative ionization mode by monitoring the fragmentation of *m*/*z* 353.00–191.00 with collision energy at 18 V for I, *m*/*z* 311.00–179.00 (23 V) for II, *m*/*z* 353.00–191.00 (14 V) for III, *m*/*z* 353.00–173.00 (16 V) for IV, *m*/*z* 179.00–135.00 (18 V) for V, *m*/*z* 609.00–285.00 (34 V) for VI, *m*/*z* 461.00–285.00 (24 V) for VII, *m*/*z* 447.00–285.00 (30 V) for VIII, *m*/*z* 445.00–269.00 (15 V) for IX and *m*/*z* 415.00–295.00 (24 V) for IS.

### 3.4. Plasma Sample Preparation

100 μL of plasma was removed and transferred to an appropriately labeled polypropylene tube (1.5 mL) containing 10 μL of internal standard solution (6 μg/mL IS), and then 300 μL of methanol was added, followed by shaking for 3 min using a vortex apparatus. The samples were centrifuged (15,000 rpm, 4 °C, 10 min) and 5 μL of clear supernatant was injected into LC-MS/MS system for bioanalysis.

### 3.5. Method Validation

#### 3.5.1. Selectivity

The response of co-eluting interferences was evaluated by the comparison of the chromatograms of the blank rat plasma, blank rat plasma spiked with nine analytes and IS and the rat plasma samples obtained 5 min after intravenous administration.

#### 3.5.2. Calibration Curves and Linearity

The stock solutions of the nine compounds were accurately weighed and diluted in methanol. Dilutions were prepared to make a series of working solutions. All stocks were stored at −20 °C. Calibration curves were plotted (10, 20, 50, 125, 250, 500, 1250, 2500 and 5000 ng/mL for compound II and 5, 10, 25, 62.5, 125, 250, 625, 1250 and 2500 ng/mL for the other eight components) using weighted linear regression of the ratio of analyte and IS (150 ng/mL) peak areas against the corresponding nominal concentration of the analyte. The LLOQ was defined as the detectable concentration at which the ratio of signal to noise was more than 10 (S/N > 10).

#### 3.5.3. Accuracy and Precision

Intra- and inter-day accuracy and precision were assessed by detecting QC samples using five replicates of rat samples at three concentration levels for compound II (30, 1000 and 4000 ng/mL) and the other eight components (15, 500 and 2000 ng/mL) on one or three validation days, respectively. Accuracy and precision were expressed by RE and RSD, respectively.

#### 3.5.4. Extraction Recoveries and Matrix Effects

The extraction recoveries and matrix effects of the nine components at above-mentioned three concentration levels and each concentration of five replicates, were evaluated. In brief, the extraction recoveries were determined by comparing the peak areas of the plasma samples spiked with analytes before extraction against the post-extraction spiked samples and calculated by the ratio of the peak responses, as well as the matrix effects were evaluated by comparing the peak areas of post-extraction spiked samples to those of the analytes in pure solution.

#### 3.5.5. Stability

Stability of the nine components were assessed under the condition of placing extracted plasma samples in auto-sampler for 6 h, as well as placing plasma samples within freeze-thaw three cycles and at −80 °C for 14 days. LQC, MQC and HQC samples with five replicates at each level were kept at the above conditions and analyzed against freshly prepared calibrators as the reference.

#### 3.5.6. Pharmacokinetic Experiment

Rats (*n* = 5) received a single intravenous administration of 15 times concentrated KI (containing 155.10 μg/mL of I, 3223.20 μg/mL of II, 136.80 μg/mL of III, 99.60 μg/mL of IV, 132.90 μg/mL of V, 366.60 μg/mL of VI, 1059.15 μg/mL of VII, 70.80 μg/mL of VIII and 154.35 μg/mL of IX) via the tail vein at a dose of 3.6 mL/kg of concentrated KI. After administration, the rat tail was carefully cleaned with alcohol soaked cotton balls around the injection site to remove the residual drug. Blood was collected from the cut-tail before dosing and at the following time points: 2.5, 5, 10, 15, 20, 30, 45 min and 1, 2, 3, 4, 6, 8, 12 and 24 h after dosing. Plasma was isolated from the blood samples by centrifugation and then stored at −80 °C until analysis.

### 3.6. Data processing

Noncompartmental pharmacokinetic parameters were calculated by DAS 3.0 software (Version DAS 3.2.8, Chinese Pharmacologic Society, Beijing, China). All the results are expressed as mean ± SD. A single-tailed Student’s t test was performed in the work.

## 4. Conclusions

In this study, a reliable and precise HPLC-ESI-MS/MS method was developed for the simultaneous quantification of five phenolic acids and four flavonoid glycosides in rat plasma after a single intravenous administration of KI. The phenolic acids remained unchanged for a longer period in the systemic circulation than flavonoid glycosides for administration of KI. In addition, compounds II and VII had a higher systematic exposure to rats. These pharmacokinetic results would be valuable to identify bioactive constituents, elucidate mechanisms of pharmacological actions and ADR and guide the rational clinical use of KI.

## Figures and Tables

**Table 1:** The regression equation, linearity range, R^2^ and LLOQ of nine compounds in the Kudiezi injection (KI).

Analyte	Regression Equation	Linearity Range (ng/mL)	R^2^	LLOQ (ng/mL)
I	y = 0.39x + 0.00195	5–2500	0.9993	5
II	y = 0.12x − 0.00323	10–5000	0.9995	10
III	y = 0.94x + 0.00709	5–2500	0.9994	5
IV	y = 0.45x + 0.00593	5–2500	0.9995	5
V	y = 0.65x + 0.0138	5–2500	0.9984	5
VI	y = 0.28x − 0.00115	5–2500	0.9989	5
VII	y = 0.48x + 0.0180	5–2500	0.9974	5
VIII	y = 0.62x + 0.0101	5–2500	0.9978	5
IX	y = 0.47x + 0.0198	5–2500	0.9944	5

LLOQ: lower limit of quantification.

**Table 2:** The precision and accuracy of nine compounds of KI in rat plasma.

Analyte	Concentration (ng/mL)	Intra-Day (*n* = 5)			Inter-Day (*n* = 5)		
		Detected (ng/mL)	RSD (%)	Accuracy (RE, %)	Detected (ng/mL)	RSD (%)	Accuracy (RE, %)
I	15	14.257	3.34	−4.95	13.557	5.30	−9.62
	500	500.226	4.21	0.05	449.267	9.70	−10.15
	2000	2129.731	2.80	6.49	1962.682	8.31	−1.87
II	30	27.386	7.17	−8.71	28.735	12.57	−4.22
	1000	1102.605	3.02	10.26	950.930	12.61	−4.91
	4000	4109.529	1.75	2.74	4066.613	2.37	1.67
III	15	12.039	18.97	−19.74	11.947	19.32	−20.35
	500	493.213	2.34	−1.36	482.248	3.87	−3.55
	2000	2041.617	3.64	2.08	2064.230	5.27	3.21
IV	15	13.395	12.71	−10.70	12.291	11.74	−18.06
	500	511.821	0.45	2.36	487.806	5.44	−2.44
	2000	2132.444	4.23	6.62	2044.840	6.19	2.24
V	15	12.376	10.20	−17.49	12.074	12.55	−19.51
	500	492.319	1.74	−1.54	450.435	14.46	−9.91
	2000	2020.753	2.07	1.04	1780.322	15.39	−10.98
VI	15	13.368	11.23	−10.88	15.466	20.49	3.10
	500	471.679	5.01	−5.66	439.213	8.93	−12.16
	2000	1936.785	3.56	−3.16	1915.598	6.16	−4.22
VII	15	14.459	15.81	−3.61	14.403	18.32	−3.98
	500	491.497	4.83	−1.70	449.924	9.11	−10.02
	2000	1922.895	2.69	−3.86	1826.993	6.82	−8.65
VIII	15	12.582	16.20	−16.12	15.470	17.60	3.13
	500	511.716	4.95	2.34	508.816	6.78	1.76
	2000	2046.100	2.81	2.30	2055.827	6.21	2.79
IX	15	12.630	4.75	−15.80	14.874	18.46	−0.84
	500	488.049	3.54	−2.39	445.026	8.28	−10.99
	2000	1758.415	4.41	−12.08	1686.240	5.63	−15.69

RSD: expressed as relative standard deviation. RE, %: expressed as relative error.

**Table 3:** The extraction recoveries and matrix effects of nine compounds of KI in rat plasma.

Analyte	Concentration (ng/mL)	Extraction Recoveries (*n* = 5)		Matrix Effects (*n* = 5)	
		Mean ± SD (%)	RSD (%)	Mean ± SD (%)	RSD (%)
I	15	79.04 ± 12.01	15.20	86.47 ± 3.41	3.94
	500	102.15 ± 11.92	11.67	87.12 ± 7.99	9.17
	2000	110.44 ± 7.70	6.97	80.66 ± 4.65	5.77
II	30	77.25 ± 14.21	18.40	>120%	17.84
	1000	93.55 ± 10.96	11.72	>120%	13.14
	4000	92.50 ± 9.94	10.75	>120%	2.98
III	15	80.93 ± 12.44	15.38	97.01 ± 17.97	18.53
	500	99.11 ± 14.40	14.52	97.56 ± 12.02	12.32
	2000	108.07 ± 9.19	8.50	98.09 ± 5.45	5.56
IV	15	80.22 ± 13.97	17.42	96.01 ± 10.35	10.78
	500	95.06 ± 10.15	10.68	112.54 ± 13.71	12.18
	2000	106.11 ± 13.71	12.92	109.26 ± 7.08	6.48
V	15	96.72 ± 14.92	15.42	87.40 ± 7.05	8.06
	500	104.88 ± 7.39	7.05	83.38 ± 5.72	6.86
	2000	118.98 ± 10.42	8.75	76.62 ± 5.68	7.41
VI	15	71.81 ± 13.33	18.57	105.58 ± 18.19	17.23
	500	79.45 ± 7.94	9.99	114.20 ± 7.96	6.97
	2000	80.38 ± 7.90	9.83	120.83 ± 7.51	6.22
VII	15	80.69 ± 8.93	11.06	112.26 ± 6.54	5.82
	500	81.63 ± 11.42	13.98	117.54 ± 7.13	6.07
	2000	90.05 ± 7.69	8.54	117.84 ± 8.99	7.63
VIII	15	68.01 ± 10.65	15.65	104.51 ± 8.42	8.06
	500	87.87 ± 5.70	6.48	107.99 ± 2.42	2.24
	2000	98.45 ± 8.54	8.67	113.43 ± 5.89	5.19
IX	15	108.11 ± 16.38	15.15	104.55 ± 17.10	16.35
	500	106.88 ± 13.60	12.72	111.22 ± 8.41	7.56
	2000	105.23 ± 9.64	9.16	109.25 ± 3.98	3.64

SD: expressed as standard deviation. RSD: expressed as relative standard deviation.

**Table 4:** The stability of nine compounds of KI in rat plasma.

Analyte	Spiked (ng/mL)	6 h in Auto-Sample		Freeze-Thaw Stability (Three Cycles)		Long-Term Stability (−80 °C, 14 Days)	
			RSD (%)	RE (%)	RSD (%)	RE (%)	RSD (%)	RE (%)
I	15	9.43	−15.03	3.59	−16.92	10.78	−10.71
	500	4.47	−14.93	8.33	−8.67	2.88	−1.12
	2000	8.69	−5.26	4.79	−2.66	4.79	−3.42
II	30	3.11	−12.49	6.28	−17.36	10.76	1.30
	1000	4.94	−9.25	6.92	2.66	4.22	−5.34
	4000	2.81	3.63	1.42	3.72	5.60	−10.05
III	15	9.45	−7.40	6.24	−18.29	16.40	−1.71
	500	10.09	−14.11	4.17	−5.77	2.36	−1.20
	2000	12.51	−2.78	3.39	2.98	4.79	2.07
IV	15	4.48	−14.16	5.18	−14.73	14.00	−10.44
	500	5.79	−15.00	4.14	−0.15	3.18	−1.40
	2000	8.89	6.12	1.62	6.55	5.67	2.14
V	15	5.96	−18.04	8.47	−13.66	10.02	−17.21
	500	6.18	−14.10	5.15	−2.10	5.63	−5.99
	2000	13.85	−11.88	5.58	−7.76	2.65	7.83
VI	15	7.10	−14.11	5.94	−13.29	8.77	−0.68
	500	2.16	−14.25	10.28	−3.78	0.89	2.31
	2000	6.52	0.23	7.58	9.34	6.84	−5.24
VII	15	10.02	−7.74	10.41	−10.80	6.63	−2.91
	500	6.91	−12.46	4.02	−1.08	1.86	−0.26
	2000	6.73	5.65	5.59	9.76	5.75	8.01
VIII	15	8.53	−12.91	13.37	−11.91	7.15	−1.72
	500	7.08	−8.04	5.68	0.67	0.69	0.81
	2000	4.95	0.65	2.75	3.11	3.21	11.47
IX	15	6.45	−13.77	18.42	−13.08	7.99	−2.73
	500	5.34	−1.56	0.78	0.53	0.77	−0.28
	2000	5.41	7.78	4.93	3.36	5.33	5.33

RSD: expressed as relative standard deviation. RE, %: expressed as relative error.

**Table 5:** The pharmacokinetic parameters of thirteen compounds in rat plasma after intravenous administration of DI with high and low dosages (Mean ± SD, *n* = 5).

Analyte	*t*_1/2_ (h)	AUC_0-t_ (h·ng/mL)	AUC_0-∞_ (h·ng/mL)	V_d_ (L/kg)	Cl (L/h/kg)	MRT_0-t_ (h)	MRT_0-∞_ (h)
I	2.22 ± 1.15	169.99 ± 55.71	209.20 ± 75.65	8.22 ± 2.51	2.99 ± 1.13	2.29 ± 1.65	3.94 ± 2.71
II	3.93 ± 0.47	2759.11 ± 399.66	3077.63 ± 482.58	22.10 ± 6.21	3.85 ± 0.62	3.84 ± 1.84	6.25 ± 2.83
III	3.36 ± 0.97	155.43 ± 23.39	199.84 ± 37.15	11.96 ± 3.10	2.53 ± 0.43	3.30 ± 0.99	5.93 ± 1.39
IV	2.92 ± 1.13	159.56 ± 39.37	209.31 ± 54.02	7.03 ± 1.29	1.81 ± 0.49	2.72 ± 1.32	5.07 ± 2.17
V	6.09 ± 0.90	145.82 ± 40.84	236.56 ± 43.58	17.92 ± 1.44	2.08 ± 0.42	4.09 ± 1.36	9.75 ± 1.70
VI	0.23 ± 0.12	163.85 ± 8.34	173.37 ± 14.97	2.51 ± 1.13	7.66 ± 0.71	0.20 ± 0.08	0.28 ± 0.15
VII	0.66 ± 0.20	671.81 ± 106.60	680.45 ± 108.60	5.30 ± 1.21	5.73 ± 0.97	0.25 ± 0.11	0.30 ± 0.12
VIII	0.13 ± 0.02	29.49 ± 2.38	31.01 ± 2.09	1.51 ± 0.35	8.25 ± 0.54	0.09 ± 0.02	0.11 ± 0.02
IX	0.07 ± 0.01	55.74 ± 8.50	56.11 ± 8.49	1.00 ± 0.24	10.07 ± 1.43	0.09 ± 0.01	0.09 ± 0.01
